# *Aggregatibacter actinomycetemcomitans* Dispersin B: The Quintessential Antibiofilm Enzyme

**DOI:** 10.3390/pathogens13080668

**Published:** 2024-08-07

**Authors:** Jeffrey B. Kaplan, Svetlana A. Sukhishvili, Miloslav Sailer, Khalaf Kridin, Narayanan Ramasubbu

**Affiliations:** 1 Laboratory for Skin Research, Institute for Medical Research, Galilee Medical Center, Nahariya 2210001, Israel; dr_kridin@hotmail.com; 2 Department of Materials Science and Engineering, Texas A&M University, College Station, TX 77843, USA; svetlana@tamu.edu; 3 Kane Biotech Inc., Winnipeg, MB R3T 6G2, Canada; msailer@kanebiotech.com; 4 The Azrieli Faculty of Medicine, Bar-Ilan University, Safed 1311502, Israel; 5 Department of Oral Biology, Rutgers School of Dental Medicine, Newark, NJ 07103, USA; ramasun1@sdm.rutgers.edu

**Keywords:** biofilm matrix, biomaterial coating, DspB, EPS, exopolysaccharide, extracellular DNA, eDNA, matrix-degrading enzyme, PIA, PNAG, *Staphylococcus aureus*, *Staphylococcus epidermidis*

## Abstract

The extracellular matrix of most bacterial biofilms contains polysaccharides, proteins, and nucleic acids. These biopolymers have been shown to mediate fundamental biofilm-related phenotypes including surface attachment, intercellular adhesion, and biocide resistance. Enzymes that degrade polymeric biofilm matrix components, including glycoside hydrolases, proteases, and nucleases, are useful tools for studying the structure and function of biofilm matrix components and are also being investigated as potential antibiofilm agents for clinical use. Dispersin B is a well-studied, broad-spectrum antibiofilm glycoside hydrolase produced by *Aggregatibacter actinomycetemcomitans*. Dispersin B degrades poly-*N*-acetylglucosamine, a biofilm matrix polysaccharide that mediates biofilm formation, stress tolerance, and biocide resistance in numerous Gram-negative and Gram-positive pathogens. Dispersin B has been shown to inhibit biofilm and pellicle formation; detach preformed biofilms; disaggregate bacterial flocs; sensitize preformed biofilms to detachment by enzymes, detergents, and metal chelators; and sensitize preformed biofilms to killing by antiseptics, antibiotics, bacteriophages, macrophages, and predatory bacteria. This review summarizes the results of nearly 100 in vitro and in vivo studies that have been carried out on dispersin B since its discovery 20 years ago. These include investigations into the biological function of the enzyme, its structure and mechanism of action, and its in vitro and in vivo antibiofilm activities against numerous bacterial species. Also discussed are potential clinical applications of dispersin B.

## 1. Introduction

Biofilms are densely packed communities of microorganisms, enclosed in a self-synthesized extracellular polymeric matrix, growing attached to a tissue or surface [1]. Biofilm is the primary mode of growth for microbes in most natural, industrial, and clinical environments. Biofilms exhibit a high tolerance to exogenous stress, and treatment of biofilms with biocides is usually ineffective at eradicating them [2]. Biofilms create many problems, ranging from industrial corrosion and biofouling to chronic and nosocomial infections.

Various antibiofilm strategies are currently being investigated. These include biomaterial surface modifications, quorum-sensing inhibitors, quorum-quenching enzymes, bacteriophages and phage-derived enzymes, and biofilm-matrix-degrading enzymes [3]. The biofilm matrix is a good target for antibiofilm agents because, unlike cells buried deep within the biofilm colony, the biofilm matrix is highly accessible to the outside environment and is inherently porous [4]. Agents that degrade or destabilize the biofilm matrix can inhibit biofilm formation or promote the detachment of established biofilm colonies [3]. Once the biofilm colony is dispersed, the cells exhibit increased sensitivity to killing by biocides and host defenses [2].

Numerous biofilm-matrix-degrading enzymes have been described [5,6,7]. These include various glycoside hydrolases, proteases, and nucleases, which degrade the polysaccharide, protein, and nucleic acid components of the biofilm matrix, respectively. These biopolymers have been shown to mediate fundamental biofilm-related phenotypes including surface attachment, intercellular adhesion, and biocide resistance [4]. The advantages of biofilm-matrix-degrading enzymes are that they exhibit broad-spectrum activity and they exert little or no selection pressure because they generally do not kill bacteria or inhibit their growth. The disadvantage of these enzymes is that they release microbial cells from the biofilm that can spread and cause infections at distant sites or elicit a hyper-inflammatory or hyper-immunogenic response [6]. Therefore, biofilm-matrix-degrading enzymes may be more useful for biofilm prevention rather than for the treatment of established biofilms, or they may need to be used in combination with antimicrobial agents to minimize these risks.

The glycoside hydrolase dispersin B is one of the best-studied biofilm-matrix-degrading enzymes. Dispersin B hydrolyzes poly-β(1,6)-*N*-acetylglucosamine (PNAG), a biofilm matrix polysaccharide that plays a role in surface attachment, biofilm formation, and biocide resistance in a wide range of Gram-negative and Gram-positive pathogens [8]. This review describes the initial discovery and characterization of dispersin B from *Aggregatibacter actinomycetemcomitans*, as well as subsequent studies on its structure and mechanism of action. Also highlighted are numerous studies demonstrating that dispersin B exhibits broad-spectrum antibiofilm activity against more than 25 phylogenetically diverse bacterial species in vitro and in vivo. Some potential clinical applications of dispersin B, such as medical device coatings, topical wound gels, and combination products, will also be discussed.

**Figure 1:**
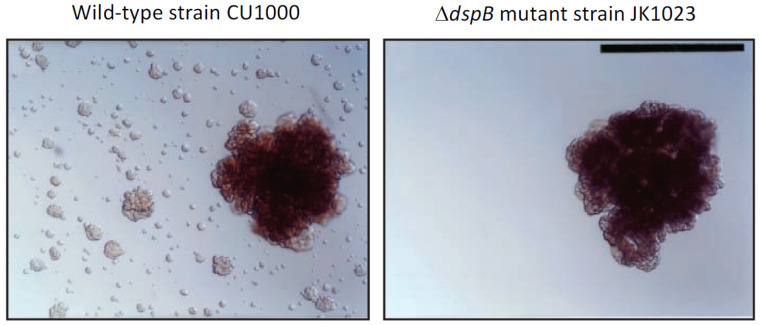
Dispersal of isolated *Aggregatibacter actinomycetemcomitans* biofilm colonies growing on the surface of polystyrene Petri dishes: (**left panel**) wild-type strain CU1000; (**right panel**) Δ*dspB* mutant strain JK1023. Satellite colonies surrounding the dispersed CU1000 biofilm colony were absent in the JK1023 culture. Photos were taken 3 d after inoculation. Scale bar = 1 mm. Image from [12].

**Discovery of dispersin B:** The Gram-negative, non-motile periodontopathogen *A. actinomycetemcomitans* forms extremely tenacious biofilms on abiotic surfaces such as plastic and glass in vitro [9]. Its adherence is so strong that the broth shows no turbidity, removal of cells from the culture vessel surface by vortex agitation is negligible, and aliquots of medium taken from the culture are often sterile upon subculture. This remarkable phenotype makes *A. actinomycetemcomitans* a useful model for studying the process of biofilm dispersal, because cells that detach from mature biofilm colonies adhere tightly to the surface of the culture vessel and form independent daughter biofilm colonies that can be visualized and enumerated (Figure 1, left panel). Screening a transposon mutant library of *A. actinomycetemcomitans* strain CU1000 identified five mutant strains that were defective in biofilm dispersal (Figure 1, right panel). The transposons in three mutant strains inserted into genes required for lipopolysaccharide O-side-chain biosynthesis [10]; the transposon in one mutant strain inserted into *ptsI*, which encodes a regulator of sugar uptake and catabolite repression (J.B. Kaplan, unpublished results); and the transposon in one mutant strain (designated JK1023) inserted into a novel gene encoding a putative β-hexosaminidase enzyme [11]. The gene disrupted in the mutant strain JK1023 was named *dspB*, and the protein that it encodes was named dispersin B. A plasmid carrying a wild-type *dspB* gene restored the ability of JK1023 biofilm colonies to disperse [11].

**Biological functions of dispersin B:** Although the *A. actinomycetemcomitans dspB* mutant strain JK1023 exhibited a severe biofilm dispersal defect in broth, it exhibited wild-type surface attachment and biofilm formation phenotypes (Figure 1). Strain JK1023 also produced colonies on agar that had a hard texture and were extremely difficult to remove from the agar surface. In test tubes, JK1023 cells aggregated and settled to the bottom of the tube much more rapidly than cells of the wild-type strain CU1000 [11]. These phenotypes demonstrate that dispersin B decreases the intercellular adhesion of *A. actinomycetemcomitans* in vitro. Stacy et al. [13] constructed a Δ*dspB* mutation in a different *A. actinomycetemcomitans* parental strain (strain 624). They confirmed that dispersin B promotes biofilm dispersal in vitro and further demonstrated that dispersin-B-mediated biofilm dispersal is triggered by oxygen and H_2_O_2_. In a murine abscess model, the *A. actinomycetemcomitans* 624 Δ*dspB* mutant strain established similar single-species infections compared to the wild-type strain, but upon co-infection with *Streptococcus gordonii* the 624 Δ*dspB* mutant strain formed larger cell aggregates than those formed by the wild-type strain, and these aggregates were located closer to *S. gordonii* aggregates than those of the wild-type strain. These findings suggest that dispersin B can modulate the spatial organization of cells within multi-species biofilms in vivo.

Zhang et al. [14] constructed a Δ*dspB* mutation in *Actinobacillus pleuropneumoniae* strain 4074, a swine pathogen that produces an orthologue of *A. actinomycetemcomitans* dispersin B [15]. The *A. pleuropneumoniae* Δ*dspB* mutant strain exhibited increased autoaggregation and biofilm formation in vitro, phenotypes that were not evident when a wild-type *dspB* gene was supplied on a plasmid. These findings confirm that dispersin B modulates bacterial intercellular adhesion and biofilm formation in different species in vitro.

**Figure 2:**
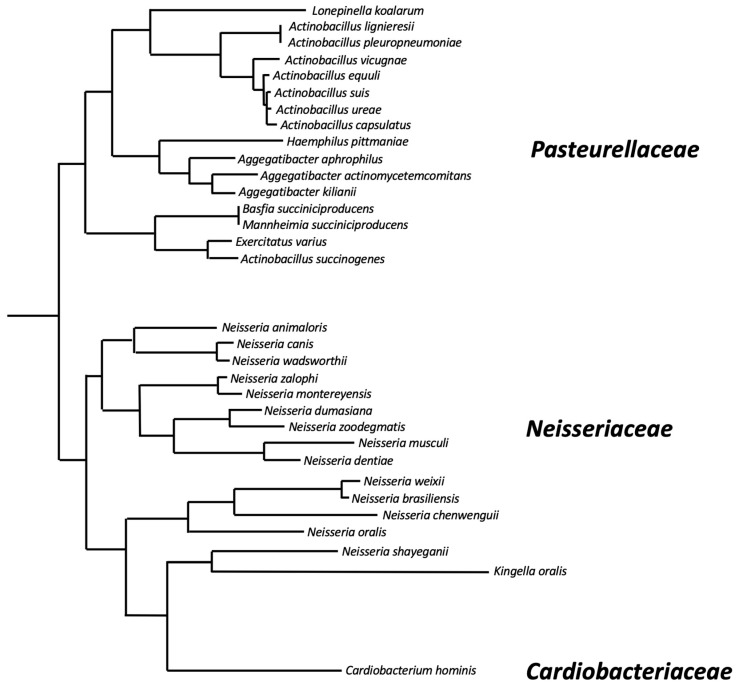
Phylogenetic relatedness of dispersin B homologues based on pairwise alignments of the amino acid sequences listed in Table 1. The alignment was generated using ClustalW, and the phylogenetic tree was generated using FastTree software. Lacto-*N*-biosidase from *Lactococcus lactis* (GenBank accession number AGY45663.1) was used as an outgroup to locate the root of the tree. Horizontal branch lengths are proportional to the number of amino acid differences in the pairwise alignments. Bacterial families are indicated on the right.

**The *dspB* gene:** The *A. actinomycetemcomitans dspB* gene encodes a protein of 381 amino acids that includes a 20-amino-acid N-terminal signal sequence that is cleaved upon secretion outside the cell. The genomes of at least 32 different bacterial species contain genes that exhibit >50% identity to *A. actinomycetemcomitans dspB* at the amino acid level (Table 1 and Figure 2) These include 16 species of *Pasteurellaceae*, 15 species of *Neisseriaceae*, and *Cardiobacterium hominis* (family *Cardiobacteriaceae*). *Pasteurellaceae* and *Neisseriaceae* have been found on the mucosal surfaces of the upper respiratory tracts of vertebrates and are often opportunistic pathogens [16]. *C. hominis* is a normal human oral and upper respiratory commensal that is rarely a cause of endocarditis [17]. The phylogeny of *dspB* homologues was congruent with the phylogenetic tree at the species level (Figure 2), suggesting that *dspB* emerged in an ancestor of these three bacterial families. All of the amino acid residues that play a critical role in *A. actinomycetemcomitans* dispersin B substrate hydrolysis (Arg27, Asp183, Glu184, Glu332; see below), as well as the three tryptophan residues at positions 216, 237, and 330 that line part of the substrate-binding pocket, were conserved in 31 of the 32 *dspB* homologues analyzed. Only the *Kingella oralis* homologue has substitutions at these critical positions (Arg27His, Glu184Ala, Trp237His, Trp330Glu, Glu332Asp). This suggests that most *dspB* homologues have the potential to encode functional dispersin B enzymes. Differences in the lengths of the predicted proteins result from N- or C-terminal extensions in the sequences of some species. Only small insertions/deletions of 1-4 amino acids are present within the core region of the protein.

Several studies have investigated the transcriptional regulation of *A. actinomycetemcomitans dspB*, which is flanked by an upstream promoter sequence and a downstream rho-independent transcription terminator sequence and does not appear to be part of an operon. Stacy et al. [18] analyzed the transcriptome of *A. actinomycetemcomitans* strain VT1169 during oxic and anoxic growth using DNA microarrays. They found that *dspB* transcription was induced by oxygen. They also cloned the *dspB* promoter upstream of a *lacZ* reporter gene and then introduced the *dspB*-*lacZ* reporter gene into *A. actinomycetemcomitans* strains 624 and VT1169. When grown as colony biofilms, both reporter strains exhibited significant β-galactosidase activity under oxic conditions but little activity under anoxic conditions. Interestingly, *dspB* induction in both strains could be mitigated by exogenously added catalase or a mutation in *oxyR* which encodes a transcriptional regulator. These findings indicate that *dspB* transcription is activated during growth with oxygen in an OxyR-dependent manner, and that the activating factor is likely H_2_O_2_. Using these same two *dspB*-*lacZ* reporter strains, Stacy et al. [18] showed that transcription of *dspB* was increased >5-fold upon iron restriction. This induction was abolished when FeSO_4_ was added to the medium. Furthermore, *dspB* transcription was increased >30-fold in a Δ*fur* mutant under the same conditions, confirming that the *dspB* promoter is regulated by iron and Fur. Other studies [19,20] showed that postbiotic compounds produced by lactic acid bacteria can modulate *dspB* expression and biofilm formation in *A. actinomycetemcomitans*, although more studies are needed to determine the mechanism of action and clinical utility of such compounds.

## 2. Production of Recombinant Dispersin B

**Production of recombinant dispersin B in *Escherichia coli*:** Kaplan et al. [11] constructed a plasmid (pRC1) that carries a gene encoding amino acids 21-381 of *A. actinomycetemcomitans* CU1000 dispersin B, fused to a 32-amino-acid C-terminal tail containing a hexahistidine metal-binding site and a thrombin protease cleavage site that can be used to cleave the C-terminal tail from the hybrid protein. This gene was located downstream from an IPTG-inducible *tac* promoter. *E. coli* strain BL21(DE3) was transformed with pRC1, induced with IPTG, and the protein was purified using Ni^2+^-affinity chromatography. After cleavage with thrombin, the purified protein migrated with the expected molecular mass of 41.5 kDa. The yield of purified dispersin B was 10 mg/L of culture. Ramasubbu et al. [21] constructed a similar plasmid (pRC3) that encodes amino acids 21-381 of CU1000 *dspB*, fused directly to a hexahistidine metal-binding C-terminal tail to facilitate crystallization. When expressed from an IPTG-inducible *tac* promoter on a plasmid and purified by Ni^2+^-affinity chromatography, this construct yielded up to 60 mg/L of dispersin B. Yakamdawala et al. [22] engineered a *dspB* gene devoid of the trinucleotide ACA. This was accomplished by silently and consecutively mutating each of the 14 occurrences of ACA in the wild-type *dspB* gene using PCR. Previous studies showed that mRNA transcripts lacking ACA sequences are protected from degradation by MazF, a sequence-specific endoribonuclease produced by *E. coli*. Expression of ACA-less *dspB* in *E. coli* strain Tuner(DE3)pLacI generated 236 mg/L of dispersin B versus 133 mg/L for wild-type *dspB* when expressed from a T7 promoter. Gökçen et al. [23] reported a dispersin B yield of about 60 mg/L when a codon-optimized *dspB* gene was cloned downstream from a tetracycline promoter/operator, transformed into *E. coli*, induced with anhydrotetracycline, and purified by Ni^2+^-affinity chromatography. In addition, Zeng et al. [24] reported that hexahistidine-tagged dispersin B purified on Ni^2+^ ion-chelated magnetic nanoparticles exhibited higher purity and activity than protein purified on conventional Ni^2+^-affinity columns.

**Production of recombinant dispersin B in tobacco:** Tobacco expression systems offer several advantages over *E. coli*, including lower costs, higher yields, and simplified downstream processing. Opdensteinen [25] expressed a codon-optimized, hexahistidine-tagged *A. actinomycetemcomitans dspB* gene in *Nicotiana tabacum* BY2 cells and *N. benthamiana* plants. *N. benthamiana* is a close relative of *N. tabacum* that is commonly used for “pharming” of recombinant proteins for clinical use. The recovery of dispersin B in planta was 75%, its purity was 96%, and a yield of up to 164 mg/kg of plant tissue was reported. These values were equivalent to those achieved in *E. coli*, suggesting that scalable purification of dispersin B in tobacco is feasible.

## 3. Dispersin B’s Structure and Mechanism of Action

*A. actinomycetemcomitans* dispersin B was crystalized using the hanging-drop vapor diffusion technique, and its 3D structure in complex with a glycerol molecule and an acetate ion at the active site was solved and refined to a resolution of 2.0 Å using the automated structure solution pipeline autoSHARP [21]. Dispersin B is a monomeric enzyme whose primary amino acid structure corresponds to that of the glycoside hydrolase family 20 group of enzymes (CAZY GH_20). This family comprises diverse β-hexosaminidases produced by both prokaryotes and eukaryotes, as well as lacto-*N*-biosidase (EC 3.2.1.14), an enzyme involved in the degradation of human milk oligosaccharides in the gut microbiota of breast-fed infants.

**Figure 3:**
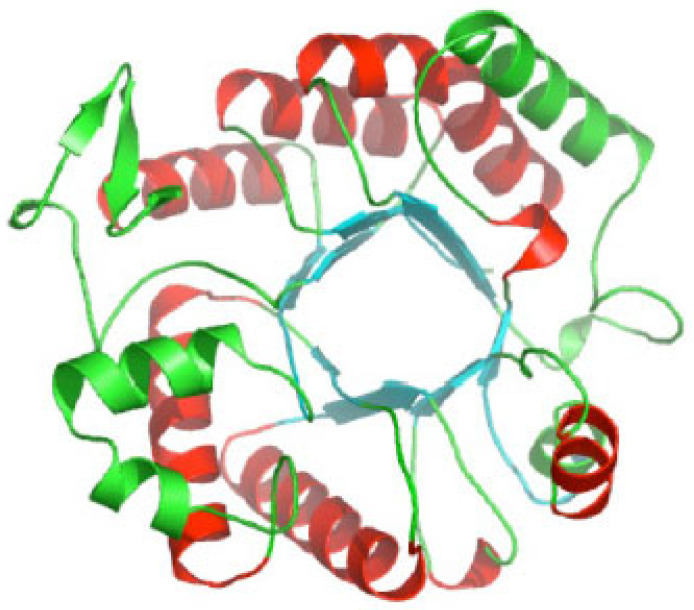
Ribbon diagram of *A. actinomycetemcomitans* dispersin B; α-helices are colored red and green; β-strands are colored blue. Image source: Wikimedia Commons.

**Figure 4:**
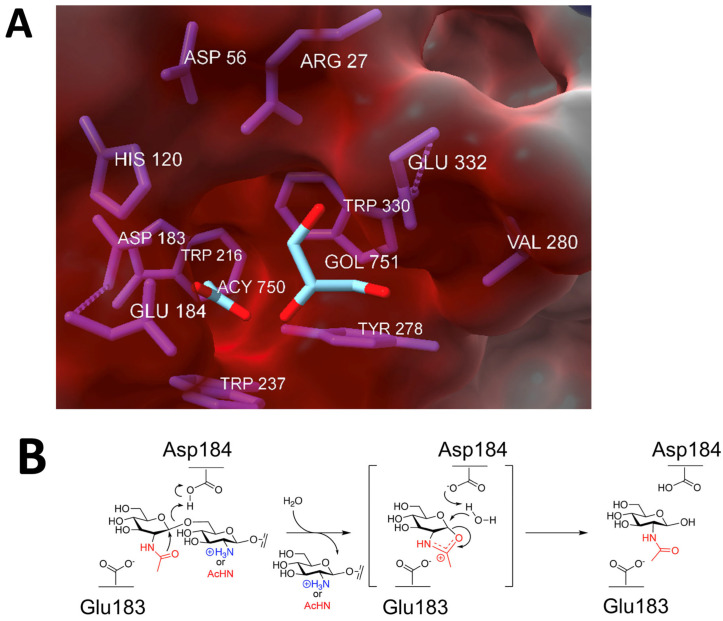
Dispersin B’s active site and mechanism of action: (**A**) Electrostatic surface potential at the active site showing the negatively charged amino acids (Asp56, Asp183, Glu184, Glu332), which create a shallow anionic region in the catalytic pocket. The size of the pocket is approximately 12 Å. GOL, glycerol; ACY, acetate. Figure generated using ChimeraX [29]. (**B**) Substrate hydrolysis mechanism proposed for dispersin B and other glycoside hydrolase family 20 hexosaminidases. In this substrate-assisted mechanism, Glu184 acts as the acid/base. The nucleophile is the *N*-acetyl group of the substrate, which is assisted by Asp183. Both exo- (dPNAG) and endoglycosidic (PNAG) cleavage are shown, where the leaving group is either deacetylated or acetylated, respectively. A suitably positioned Asp183 helps stabilize the oxazolium ion in the transition state. Figure generated using ChemDraw (PerkinElmer).

Like all glycoside hydrolase family 20 enzymes, dispersin B adopts a TIM barrel protein fold consisting of eight α-helices and eight parallel β-strands that alternate along the polypeptide backbone (Figure 3). The active site of the enzyme is a large central cavity at the center of the TIM barrel that exhibits a negative electrostatic potential due to the presence of a number of polar acidic residues that are also conserved in other β-hexosaminidases (Figure 4A). Trp216 and Trp330 form the floor of the 12 Å deep substrate-binding pocket where the hexose ring binds. Asp183 and Glu184 are the catalytic residues that are conserved in all glycoside hydrolase family 20 enzymes [26,27,28].

Evidence suggests that dispersin B utilizes a substrate-assisted mechanism, commonly referred to as the double-displacement retaining mechanism, similar to other β-hexosaminidases (Figure 4B). A unique feature of this mechanism is the participation of the acetamido group of the substrate, which provides anchimeric assistance and acts as the nucleophile while a suitably juxtaposed amino acid residue acts the acid/base. This mechanism was confirmed using biochemical analyses of native dispersin B enzymes with different substrates, as well as mutational analyses [27,30,31,32,33,34]. In this mechanism, the active site residue Asp183 binds to the *N*-acetyl group of PNAG, and Glu184 serves as the catalytic acid/base (Figure 4B). Asp183 may also help stabilize the positive charge that develops in the oxazoline transition state (Figure 4B) or help distort the substrate to direct the 2-acetamido group toward the anomeric carbon [28]. Proteins with Asp183Asn and Glu184Gln mutations exhibited >10,000-fold and >70-fold decreased activity, respectively, compared to the wild-type enzyme, irrespective of the substrate used for hydrolysis. A mutation in another acidic residue located near the catalytic residues (Glu332) exhibited 2000-fold lower activity than the native enzyme. Glu332 may provide stabilization in the transition state while the terminal glucosamine is undergoing conformational changes [27]. Mutations in Asp147 and Asp245, which are also located in the anionic pocket near the active site, also exhibited decreased enzyme activity. These residues may play a role in recognition of the cationic PNAG substrate. Four aromatic amino acid residues (Tyr187, Tyr278, Trp237, Trp330) line the hydrophobic substrate-binding pocket, where they bind to and orient the PNAG substrate. As expected, mutations in these residues exhibited 5–2400-fold less activity that the wild-type enzyme. In addition to these acidic and aromatic amino acid residues, all β-hexosaminidases have a conserved arginine that is involved in substrate binding at the active site, equivalent to Arg27 of *A. actinomycetemcomitans* dispersin B. Enzymes with Arg27Lys and Arg27Ala mutations exhibited 2400-fold and >1700-fold reductions in activity, respectively. Overall, these mutational studies confirm that dispersin B utilizes the same substrate-assisted mechanism as that utilized by other glycoside hydrolase family 20 enzymes.

All PNAG exopolysaccharides have been shown to be post-translationally modified by partial deacetylation (ca. 15–20%), which is critical for PNAG-dependent biofilm formation [8]. Dispersin B exhibits both exo- and endoglycosidase activity against PNAG, depending on the nature of the substrate [27,31,32,35]. Dispersin B exhibits greater activity against fully deacetylated PNAG (dPNAG) than against fully acetylated PNAG. Thus, the mechanism of action of dispersin B evidently depends on different patterns of deacetylation [35,36]. Studies utilizing site-directed mutagenesis and synthetic PNAG oligosaccharides demonstrated that the increased rate of hydrolysis for dPNAG was mediated by interaction of the glucosamine residues of dPNAG with Asp147 and Asp242, which are located in a shallow anionic groove adjacent to the catalytic pocket [36,37]. Dispersin B containing an Asp242Asn mutation was highly deficient in endoglycosidase activity while maintaining exoglycosidase activity. These findings suggest that dispersin B exhibits endoglycosidic cleavage against dPNAG due to the absence of an acetamido group on dPNAG. The exhibition of both exo- and endoglycosidic activity by dispersin B might be critical during biofilm formation and dispersal, since this would catalyze the hydrolysis of both PNAG and dPNAG in an efficient manner.

## 4. Dispersin B as a Tool for Studying Biofilms

**Figure 5:**
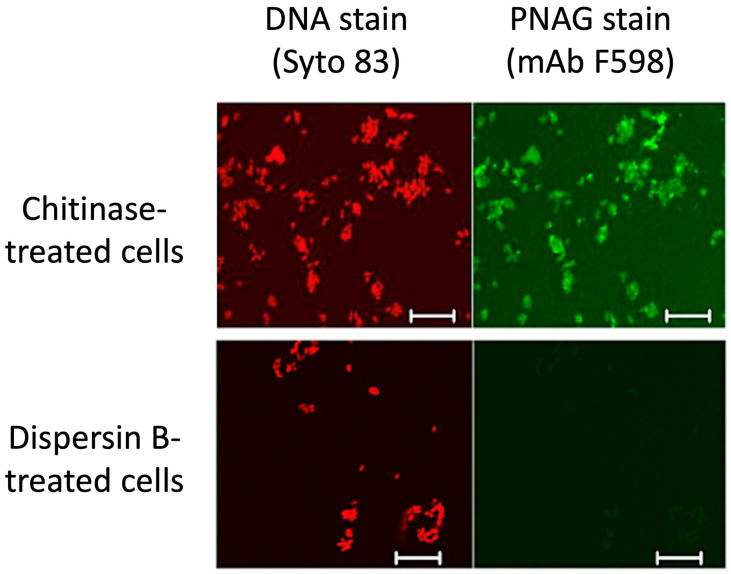
Confocal microscopic analysis of PNAG expression by *Y. pestis* strain KIM6+ grown at 28 °C overnight on Congo red agar. After treatment of bacterial cells with either chitinase (**top panels**) or dispersin B (**bottom panels**), cells were stained with Syto 83 to visualize DNA (red) and Alexa Fluor 488-conjugated mAb F598 to detect PNAG (green). Bars = 10 µm. Figure from Yoong et al. [40].

**Dispersin B as a probe for PNAG production:** PNAG has been identified as a highly conserved surface polysaccharide produced by diverse bacterial, fungal, and protozoal pathogens [38,39]. However, PNAG is difficult to isolate and purify because it is usually produced at low levels and is tightly bound to the cell surface. An alternative method for detecting PNAG is fluorescence confocal microscopy using the antigen-specific human IgG1 monoclonal antibody F598 [38]. Figure 5 shows that the immunoreactivity of *Yersinia pestis* cells with mAb F598 was lost after the cells were treated with dispersin B, but not with chitinase—a related glycoside hydrolase that degrades chitin, a polymer of β(1,4)-linked *N*-acetylglucosamine residues [40]. This same dispersin-B-induced loss of immunoreactivity with mAb F598 was observed in *Bacillus subtilis* [41] and several other prokaryotic and eukaryotic pathogens [38,42,43]. These findings demonstrate that dispersin B can function as a sensitive and specific probe for PNAG.

Dispersin B is often used along with proteinase K and DNase I to investigate the composition of the biofilm matrix. For example, dispersin B, but not proteinase K or DNase I, degraded insoluble extracellular matrix components of *S. aureus* strain SH1000 [44] and strain MR10 [45], confirming that the biofilm matrix of these strains primarily contains PNAG. This is consistent with the susceptibility of these strains to detachment by dispersin B.

Eddenden et al. [46] and Eddenden and Nitz [47] leveraged the specificity of dispersin B to construct a probe (Dispersin B PNAG probe or DiPP) for monitoring and localizing PNAG production during biofilm formation. DiPP was created by mutating one amino acid in the dispersin B active site (E184), which rendered the enzyme catalytically inactive but still capable of binding to PNAG, and then fusing inactive dispersin B to green fluorescent protein (GFP-DiPP). Fluorescent imaging studies demonstrated that GFP-DiPP bound to PNAG-dependent cells and biofilms, but not to PNAG-independent cells and biofilms, thereby demonstrating the specificity of the probe for PNAG. DiPP binding experiments with the PNAG-producing *E. coli* strain MG1655 revealed a high concentration of PNAG at the bacterial cell surface, which was localized in discrete areas. These distinct areas appeared to slough from the cells and accumulate in interbacterial regions during the development of a PNAG-dependent biofilm. A helical distribution of staining was also observed, suggesting spatial organization of PNAG on the cell surface prior to biofilm formation These experiments demonstrate the potential value of a highly specific dispersin B probe for monitoring PNAG production.

**Dispersin B as a probe for PNAG function:** Several studies have used dispersin B to demonstrate that PNAG plays a role in bacterial intercellular adhesion, biofilm formation, biofilm porosity, and host cell binding. Al Laham et al. [48] found that *S. epidermidis* small-colony variants, which are sometimes associated with device infections, produced large cell aggregates when cultured under planktonic conditions. These cell aggregates were completely disintegrated by dispersin B, demonstrating that PNAG serves as an intercellular adhesin, a finding that was subsequently confirmed by indirect immunofluorescence assays with anti-PNAG antiserum. Similarly, Amini et al. [49] demonstrated that exogenously added PNAG enabled non-PNAG-producing strains of *E. coli* to form biofilms, a fact that was confirmed when dispersin B treatment abolished the activity. Ganeshnarayanan et al. [50] measured the transport of water and the cationic surfactant cetylpyridinium chloride (CPC) through *S. epidermidis* and *A. pleuropneumoniae* biofilms cultured in centrifugal filter devices. Significantly more water and CPC passed through the biofilms after treatment with dispersin B compared to the amount that passed through untreated biofilms. Similarly, significantly more water and CPC passed through *S. epidermidis* and *A. pleuropneumoniae* PNAG-mutant biofilms compared to wild-type biofilms. These findings suggest that PNAG impedes fluid convection and the transport of small molecules through biofilms. Similarly, Lin et al. [51] showed that pre-treating PNAG-expressing *S. carnosus* cells with dispersin B significantly decreased their ability to bind to human RPMI 2650 nasal epithelial cells.

**Dispersin B as a tool for eDNA extraction:** Extracellular DNA (eDNA) is an important matrix component of many bacterial biofilms, but it is sometimes difficult to isolate because it binds to other biofilm matrix components, including PNAG [52]. Wu and Xi [53] showed that when biofilms of *Acinetobacter* sp. grown in 6-well microtiter plates were pre-treated with dispersin B, they yielded more eDNA than untreated biofilms. Similarly, Wu and Xi [54] showed that dispersin B treatment significantly increased the yield of eDNA extracted from *Stenotrophomonas maltophilia* and *Acinetobacter baylyi* AC811 biofilms grown in 6-well plates. Thus, dispersin B may be a useful tool for eDNA extraction and analysis.

## 5. Modifications to Dispersin B

**Chemical modification of dispersin B:** Abdelkader et al. [55] covalently modified dispersin B with nine cyclodextrin molecules. Cyclodextrins are cone-shaped molecules that contain a hydrophobic central cavity that can bind to other hydrophobic molecules. The cyclodextrin modifications had no effect on the ability of dispersin B to detach preformed biofilms produced by four strains of *S. epidermidis*. The researchers then covalently linked ciprofloxacin to a hydrophobic adamantyl group and formed a complex between dispersin B/cyclodextrin and ciprofloxacin/adamantane to create an “all-in-one” drug delivery system that could destroy the biofilm matrix and simultaneously release the antibiotic. When tested against 24-hour-old biofilms produced by *S. epidermidis* strain 5 (a PNAG-overproducing strain) in 96-well microtiter plates, the enzyme/antibiotic complex exhibited a more than 2-log increase in biofilm eradication compared to dispersin B/cyclodextrin alone, thereby demonstrating the feasibility of this approach.

**Dispersin-B-loaded nanoparticles:** Various nanobiotechnology-based approaches for eradicating bacterial biofilms, including functionalized metallic nanoparticles, are being investigated. To this end, Liu et al. [56] created a fusion protein between dispersin B and MagR, a protein involved in responses to magnetism in *Drosophila melanogaster* that can be used as a fusion partner to functionally immobilize proteins on magnetic surfaces. MagR was fused to the C-terminus of dispersin B, expressed in *E. coli*, and purified by Ni^2+^-affinity chromatography. The dispersin B-MagR fusion protein was immobilized on Fe_3_O_4_/SiO_2_ magnetic nanoparticles and tested for its ability to detach preformed biofilms produced by *Bacillus cereus*, *Staphylococcus aureus*, and one additional staphylococcal strain in 24-well microtiter plates. The authors found that Fe_3_O_4_/SiO_2_ nanoparticles loaded with the dispersin B-MagR fusion protein detached pre-biofilms more efficiently than Fe_3_O_4_/SiO_2_ nanoparticles or the dispersin B-MagR fusion protein alone. In addition, immobilization of dispersin B-MagR on magnetic nanoparticles increased the stability of the enzyme and increased its optimal temperature from 30 °C to 37 °C. Theoretically, this system could be used to deliver dispersin B to specific sites under the function of a magnetic force. Similarly, Chen and Lee [57] fused a 12-amino-acid silver-binding peptide to the N-terminus of dispersin B in order to prepare Ag nanoparticles conjugated with dispersin B. The goal was to create an agent that could both disrupt biofilms and simultaneously kill planktonic cells released from the disrupted biofilms. Although Ag nanoparticles could not be conjugated with the dispersin B/Ag-binding peptide fusion protein because dispersin B precipitated in the presence of Ag ions, the fusion protein itself was found to detach preformed *S. epidermidis* biofilms grown on silicone sheets or glass coverslips twofold more efficiently than native dispersin B.

**Dispersin B as a medical device coating:** Implanted medical devices and wound dressings coated with dispersin B have the potential to reduce the incidence of device infections and promote wound healing. Strategies for grafting dispersin B onto solid surfaces rely on either non-covalent absorption/adsorption of the enzyme to the surface, or its covalent attachment to the surface. These strategies are designed to achieve a high local concentration of dispersin B in the vicinity of the biomaterial surface.

One example of non-covalent binding of dispersin B to biomaterials was reported by Hagan et al. [58], who adsorbed dispersin B and amikacin onto a commercially available, degradable hydrogel (VetriGel). Although these agents were successfully trapped within the hydrogel, the chemistry of the hydrogel did not support long-term retention of dispersin B, and the trapped molecules underwent rapid elution within the first 24 h. A similar approach was reported by Kaplan et al. [59], who adsorbed dispersin B onto unmodified polyurethane and Teflon catheters and showed that the coated catheters efficiently resisted biofilm formation by *S. epidermidis*. The amount of enzyme retained on the surface was not measured, although catheters that were pre-coated and dried retained their antibiofilm activity after one month of storage at 4 °C.

Additional studies quantified the adsorption of dispersin B on polyurethane disks, including those functionalized with acidic and basic groups [60]. These studies showed that coating polyurethane surfaces with dispersin B resulted in a >1 log unit reduction in *S. aureus* and *S. epidermidis* biofilms compared to the amount of biofilm formed on uncoated polyurethane. In addition, staphylococcal biofilms that were grown on dispersin-B-loaded polyurethane disks and rinsed exhibited increased sensitivity to killing by cefamandole nafate compared to biofilms grown on uncoated polyurethane disks. In a similar study, Darouiche et al. [61] showed that polyurethane central venous catheters coated with dispersin B and triclosan efficiently resisted colonization by *S. aureus*, *S. epidermidis*, *E. coli*, and *C. albicans*.

In other studies, dispersin B was trapped within a porous structure of biodegradable asymmetric membranes that were designed for wound dressing applications [62,63]. The efficiency of dispersin-B-loaded poly(3-hydroxybutyrate-co-4-hydroxybutyrate) membranes against *S. epidermidis* was modest (12% reduction) and occurred only in the case of preformed biofilms [62]. However, an improved membrane micro/nanostructure controlled by a polymeric porogen, as well as treatment of membrane surfaces with NaOH to create a surface charge, enhanced the antibiofilm activity of the membrane. Specifically, *S. epidermidis* biofilm formation was inhibited by 33%, while 26% of the preformed biofilm was destroyed [63]. By further improving the nanoporosity and efficiency of a poly(butylene-succinate-co-adipate)-based asymmetric membrane using a polymeric porogen, Bou Haidar et al. [64] showed that up to 80% of preformed *S. epidermidis* biofilms could be eradicated using this approach.

**Figure 6:**
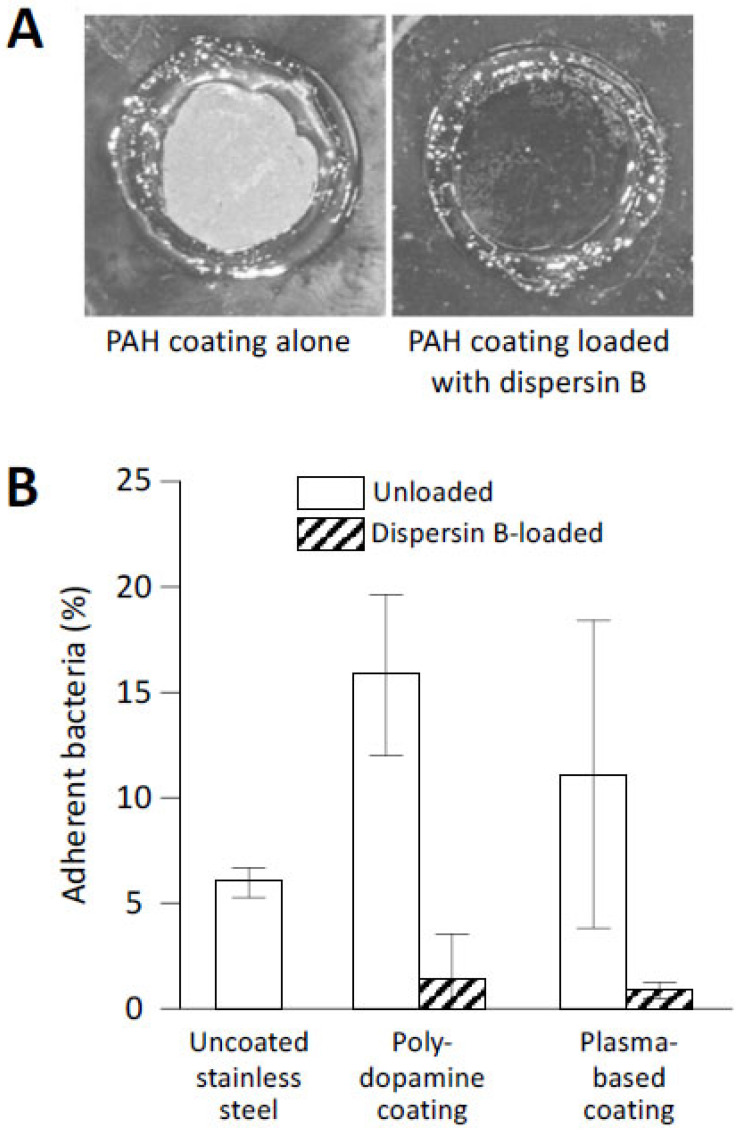
Abiotic surfaces coated with dispersin B resist *S. epidermidis* biofilm formation and surface attachment: (**A**) Biofilm formation by *S. epidermidis* strain NJ9709 on glass slides containing an ultrathin layered poly(allylamine hydrochloride) (PAH) hydrogel coating (**left panel**) or a PAH coating loaded with dispersin B (**right panel**). Bacteria were cultured inside plastic cloning cylinders (5 mm internal diameter) that were attached to the slide with high-vacuum grease. After 12 h, the biofilms were rinsed, the cloning cylinders were removed, and the slides were photographed. The rings correspond to the footprints of the cloning cylinders. The biofilm appeared as a white film on the unloaded PAH layer, which was absent on the dispersin-B-loaded PAH layer. (**B**) Attachment of *S. epidermidis* strain ATCC35984 to uncoated stainless steel disks, or to disks coated with polydopamine- or plasma-based coatings with or without grafted dispersin B. Source: (**A**) [65]; (**B**) redrawn from [66].

While a general feature of non-covalently adsorbed proteins is their tendency to desorb upon extensive dilution with a medium, controlling the nature and density of the adsorption sites can achieve strong binding of enzymes at surfaces. The latter scenario was realized by employing the layer-by-layer technique to construct surface hydrogels with a high density of basic groups, followed by trapping of dispersin B within the coatings [65]. Although dispersin B was retained within the coating only by electrostatic interactions, the coatings did not elute dispersin B in solution, were highly stable over a wide range of pH values, and maintained their antibiofilm function after a several-day-long pre-incubation in buffer solutions. These dispersin-B-loaded coatings inhibited biofilm formation by a clinical strain of *S. epidermidis* (Figure 6A). Importantly, this approach enables facile control of the amount of immobilized dispersin B by modulating the number of polymer layers in the surface hydrogels.

An alternative strategy for localized protection against biofilm growth is surface functionalization via covalent attachment of enzymes. For applications in regenerative medicine, biodegradable polyhydroxyalkanoate (PHA)-based fiber meshes were functionalized with dispersin B along with a synthetic antibacterial peptide by covalent conjugation, which was achieved by using reactive star-shaped macromolecules as an additive to a PHA solution [67]. Efficient prevention of bacterial adhesion (88%) and complete inhibition of *S. epidermidis* biofilm formation confirmed the successful presentation of the antibiofilm and antimicrobial agents at the fiber surface.

Covalent modification of solid surfaces, such as stainless steel mimicking the surfaces of biomedical implants, enables a convenient and rapid method for creating reactive surface groups using atmospheric plasma technology for the rapid modification of surfaces with protein-binding interlayers [66,68]. In one example, epoxy-rich films were created by introducing glycidyl methacrylate in the plasma, followed by covalent immobilization of dispersin B and a sulfomethoxazole-degrading enzyme (laccase). These coatings resulted in a 79–84% reduction in adherent *S. epidermidis* bacteria [68]. The atmospheric plasma technique was also used to deposit acrylic-based interlayers containing chemically reactive catechol/quinone groups on metallic surfaces for subsequent immobilization of dispersin B [66]. This biomimetic approach with both solution-adsorbed polydopamine (PDA) and plasma-based interlayers showed high antibiofilm activity against *S. epidermidis* (Figure 6B).

Faure et al. [69] used the redox and adhesive properties of 3,4-dihydroxy-L-phenylalanine (DOPA) to apply surface modifications on stainless steel surfaces for enzyme immobilization. While a cationic polyelectrolyte-bearing catechol unit that mimics the composition of adhesive proteins present in mussel feet was used to coat the surface, the capability of poly(methacrylamide)-bearing quinone groups for crosslinking with amine groups was used to prepare nanogels that could be easily deposited to stainless steel from aqueous solutions. Dispersin B containing thiol groups was then covalently anchored on the nanogels, resulting in coatings that provided long-term activity against *S. epidermidis* [69].

A different, potentially substrate-agnostic approach was recently developed that involves covalent conjugation of dispersin B to spider silk protein using the transpeptidase sortase A [70,71]. This approach is based on the ability of the silk protein to self-assemble via non-covalent interactions within a coating. The ability to use pre-assembly or post-assembly enzyme conjugation routes provides flexibility in optimizing the surface presentation of enzymes, because of the ease and efficiency of the conjugation procedure [70].

Despite the specific advantages and disadvantages of the above approaches, they all have the potential to create bioactive materials that allow local treatment of complex infections without the need for invasive procedures, and all deserve further development.

**Dispersin-B-expressing bacteria as therapeutic agents:** Several studies have investigated the use of genetically engineered bacterial strains expressing dispersin B as live therapeutics against biofilm-related infections. Garrido et al. [72] constructed an attenuated strain of *Mycoplasma pneumoniae* that secretes both dispersin B and lysostaphin, an endopeptidase that cleaves the pentaglycine crossbridge of the staphylococcal cell wall. This engineered strain significantly reduced *S. aureus* biofilm formation in polystyrene microtiter plates and on polyurethane catheters in vitro. In a murine *S. aureus* catheter infection model in vivo, mice treated with *M. pneumoniae* expressing both dispersin B and lysostaphin exhibited impaired biofilm formation compared to mice treated with *M. pneumoniae* expressing dispersin B alone. In addition, the engineered *M. pneumoniae* cells were significantly more efficient at inhibiting *S. aureus* biofilm formation than the purified dispersin B enzyme alone or supernatants from the engineered strain, suggesting that such strains have the potential to provide a continuous supply of dispersin B at infection sites. Since *M. pneumoniae* is a respiratory pathogen, these engineered strains may be useful for the treatment of biofilm-associated respiratory infections.

Ghalsasi and Sourjik [73] fused the secretion tag from *E. coli* OmpA to the N-terminus of dispersin B and transformed the hybrid gene into *E. coli* strain W3110, thereby creating a “disrupter” strain that secretes dispersin B into the surrounding medium. When tested against preformed biofilms produced by *E. coli* strain TRMG1655 in 96-well microtiter plates, the disrupter strain was found to detach 50% of the target biofilm in 12 h when induced with 100 mM IPTG. Similarly, Ragunath et al. [74] displayed dispersin B on the surface of *E. coli* by fusing dispersin B to a 290-amino-acid C-terminal region of *A. actinomycetemcomitans* Aae, an autotransporter protein involved in host cell binding. The C-terminal region of Aae inserts into the outer membrane and anchors the fusion partner in the membrane. *E. coli* cells that displayed dispersin B on their surface efficiently detached preformed *S. epidermidis* and *A. pleuropneumoniae* biofilms in a 96-well microtiter plate assay, further demonstrating the potential utility of this approach for biofilm control.

**Enzymatic bacteriophages:** Bacteriophages are being investigated as an alternative to antibiotics for the treatment of bacterial infections, including those caused by biofilms. Lu and Collins [75] engineered the lytic *E. coli*-specific phage T7 to express dispersin B intracellularly during infection so that dispersin B would be released into the extracellular environment upon cell lysis. When tested against preformed biofilms formed by *E. coli* strain TG1 on 96-peg lids, the engineered enzymatic phage reduced bacterial biofilm cell counts by ≈4.5 log units (≈99.997% removal), which was 2 log units greater than the reduction achieved with non-enzymatic phages. Schmerer et al. [76] confirmed that dispersin-B-expressing T7 phages were superior to non-enzymatic phages for eradicating *E. coli* biofilms grown for 12–16 h in 24-well microtiter plates, but they were only marginally better than non-enzymatic phages against *E. coli* biofilms grown for 7 d in silicone tubing. These studies demonstrate the feasibility of using engineered enzymatic bacteriophages as an antibiofilm strategy.

**Enzyme cocktails:** Wen et al. [77] tested different combinations of dispersin B, proteinase K, and DNase I against biofilms produced by 10 multidrug-resistant *Corynebacterium striatum* strains in 96-well microtiter plates. They found that the combination of 20 µg/mL dispersin B and 20 µg/mL proteinase K was most effective, dispersing at least 50% of the biofilm in 9/10 strains. Poilvache et al. [78] measured the ability of a tri-enzyme cocktail to detach biofilms produced by *S. aureus*, *S. epidermidis*, and *E. coli* on titanium surfaces. The enzymes were a nonspecific endonuclease from *Serratia marcescens*, an endoglucanase from *Aspergillus niger*, and dispersin B from *A. pleuropneumoniae*. The tri-enzyme combination exhibited greater biofilm-detaching activity than any of the individual enzymes against *S. epidermidis*, but the combination was not more effective than endonuclease alone against *S. aureus* or dispersin B alone against *E. coli*. Exposure of tri-enzyme-treated biofilms to antibiotics resulted in a 2–3 log unit reduction in the total CFUs compared to biofilms treated with antibiotics alone in all three species. In a similar study from the same laboratory, Ruiz-Sorribas et al. [79] measured the ability of the tri-enzyme cocktail to detach three-species biofilms formed by *S. aureus*, *E. coli*, and *Candida albicans* in 96-well microtiter plates and on glass coverslips. They found that the addition of *Bacillus subtilis* lyticase or *B. licheniformis* subtilisin A was necessary to achieve significant detachment of *C. albicans* biofilms. Pre-exposure of three-species biofilms to enzymes potentiated the activity of antimicrobials against the biofilms, including the activity of caspofungin against *C. albicans*. Waryah et al. [80] showed that despite the inferiority of dispersin B to DNase I in dispersing *S. aureus* biofilms in a 96-well microtiter plate assay, both enzymes were equally efficient in enhancing the antibacterial efficiency of tobramycin. However, a combination of these two enzymes was found to be significantly less effective in enhancing the antimicrobial efficacy of tobramycin than the individual enzymes alone. Finally, Chiba et al. [81] investigated the effects of combined RNase A and dispersin B treatment on *S. aureus* biofilm formation when grown in 96-well plates. When administered at low concentrations, neither enzyme alone dispersed the mature biofilms. However, efficient dispersal was achieved by incubation with both enzymes, even at low concentrations. Taken together, these findings suggest that combining dispersin B with other biofilm-matrix-degrading enzymes could increase their efficacy and spectrum of activity.

## 6. Antibiofilm Activities of Dispersin B against Bacteria

As outlined in Table 2, dispersin B exhibits various antibiofilm activities against more than 25 different species of Gram-negative and Gram-positive bacteria in vitro. These activities include (i) inhibition of biotic and abiotic surface attachment; (ii) inhibition of biofilm formation; (iii) detachment of preformed biofilms; (iv) inhibition of pellicle formation (biofilms at the air–liquid interface); (v) disaggregation of bacterial flocs (floating or suspended biofilms); (vi) sensitization of preformed biofilms to detachment by EDTA, SDS, proteinase K, DNase, and high-velocity water irrigation; (vii) sensitization of biofilms to killing by antibiotics (ampicillin, cefamandole nafate, ciprofloxacin, clindamycin, rifampicin, tetracycline, tobramycin, vancomycin), antiseptics (benzoyl peroxide, cetylpyridinium chloride, SDS, triclosan), antimicrobial peptides (KSL-W, LL-37, polymyxin B), bacteriophages, human macrophages, and predatory *Bdellovibrio* bacteria; and (viii) inhibition of hyphal aggregation and surface adhesion in *Streptomyces* spp. Taken together, these findings confirm that PNAG plays a role in diverse biofilm-related functions, and that dispersin B exhibits broad-spectrum antibiofilm activity.

Some studies have shown that the ability of dispersin B to inhibit biofilm formation and detach preformed biofilms depends on the shape, size, and composition of the culture vessel. For example, biofilm formation by *Cutibacterium acnes* was inhibited by dispersin B when biofilms were cultured in glass tubes, but not when cultured in 96-well polystyrene microtiter plates [99]. Similarly, dispersin B efficiently detached *A. actinomycetemcomitans* biofilms cultured in polystyrene tubes, but not in polystyrene microtiter plate wells [91]. These results may reflect differences in biofilm architecture or biofilm matrix composition resulting from differences in the culture vessel shape, culture volume, surface-to-volume ratio, or substrate material. In addition, some studies have found that dispersin B treatment appears to increase biofilm formation when the biofilm’s biomass is measured using a crystal violet binding assay. For example, Izano et al. [91] found that treatment of preformed *A. actinomycetemcomitans* biofilms cultured in 96-well polystyrene microtiter plates with dispersin B resulted in a significant increase in crystal violet binding compared to mock-treated biofilms. Similarly, Atwood et al. [122] found that biofilms formed by *S. aureus rsbU* and *sigB* mutant strains in microtiter plates bound significantly more crystal violet dye when they were cultured in dispersin-B-supplemented broth compared to the amount of bound dye in unsupplemented broth. One possible explanation for these results is that dispersin B increases the volume and porosity of the biofilm matrix, thereby allowing more crystal violet dye molecules to enter the biofilm.

Numerous studies have reported that dispersin B exhibits no bacteriostatic or bactericidal activity against a wide range of Gram-positive and Gram-negative bacteria. These results are most often reported as “data not shown”. However, LeBel et al. [106] found that dispersin B exhibited dose-dependent growth inhibition of *Solobacterium moorei* in microtiter plate wells, with approximately 50% growth inhibition at 5–50 µg/mL dispersin B. Other studies showed that dispersin B was not cytotoxic against human HEp-2 larynx carcinoma cells, human HaCaT keratinocytes, human THP-1 monocytes, human MG-63 osteoblasts, murine J774 macrophages, murine L929 fibroblasts, or sheep erythrocytes [60,64,79].

**Antibiofilm activities of dispersin B against staphylococci:** *S. aureus* has received considerable attention because it causes many serious biofilm-related infections and also forms PNAG-dependent biofilms. Nearly all *S. aureus* strains carry the *icaADBC* operon, which encodes the enzymes required for PNAG biosynthesis [8]. However, only some strains appear to rely on PNAG expression for biofilm formation in vitro and in vivo. This fact is reflected in the varied responses of *S. aureus* biofilms to dispersin B treatment. For example, Hogan et al. [107] measured the ability of dispersin B to detach 24-hour-old *S. aureus* biofilms grown in plasma-coated microtiter plate wells. They found that dispersin B at 0.125–4 µg/mL effectively detached biofilms formed by *S. aureus* strain SH1000, a methicillin-sensitive *S. aureus* (MSSA) strain, but not those formed by *S. aureus* strain JE2, a methicillin-resistant *S. aureus* (MRSA) strain. However, dispersin B at 1 µg/mL was able to sensitize both SH1000 and JE2 biofilms to killing by a combination of rifampicin and vancomycin, although the sensitization effect was significantly greater for strain SH1000 (6–7 log units) than for strain USA300 JE2 (1–2 log units). Similarly, Izano et al. [108] found that dispersin B efficiently detached preformed biofilms produced by MSSA strain SH1000 in 96-well microtiter plates, but not those produced by MRSA strain 252. However, dispersin B did not inhibit biofilm formation by either strain, and it did not sensitize MSSA strain SH1000 biofilms to killing by cetylpyridinium chloride. Asai et al. [109] found that only one of twelve *S. aureus* strains isolated from patients with catheter-related bloodstream infections was susceptible to detachment by dispersin B when cultured in 96-well microtiter plates. Instead, most strains were sensitive to detachment by proteinase K. Similarly, Sugimoto et al. [110] found that dispersin B exhibited very limited biofilm inhibition and detachment activities against a panel of 17 *S. aureus* strains (10 MSSA, 7 MRSA) isolated from hospital patients when tested in 96-well microtiter plates. In contrast, Rohde et al. [111] found that 18 out of 18 *S. aureus* strains isolated from prosthetic joint infections were efficiently detached from 96-well plates by dispersin B. These differences may reflect differences in the media, culture conditions, bacterial strains, or methods used.

Several studies have shown that dispersin B sensitizes *S. aureus* biofilms to killing by a variety of antimicrobial agents, including triclosan [59], cefamandole nafate [60], silver [103], the antimicrobial peptide KSL-W [83], and a combination of rifampicin and clindamycin [123]. In general, dispersin B appears to sensitize both MSSA and MRSA biofilms to antibiotic killing.

*S. epidermidis* is of interest because of its ability to cause biofilm-related implant infections and its high susceptibility to biofilm inhibition and detachment by dispersin B [59,113]. Unlike *S. aureus*, only some *S. epidermidis* strains carry the *icaADBC* operon. It is still unclear whether the presence of *icaADBC* in *S. epidermidis* is correlated with an increased risk of device infection. Numerous studies have shown that even low concentrations of dispersin B efficiently inhibit and detach PNAG-dependent *S. epidermidis* biofilms in vitro [61,67,82,100,109,111,112,114,115,116,117,124] and sensitize *S. epidermidis* biofilms to killing by antimicrobial agents such as cetylpyridinium chloride [50,108], silver [103], and rifampicin [118].

**Figure 7:**
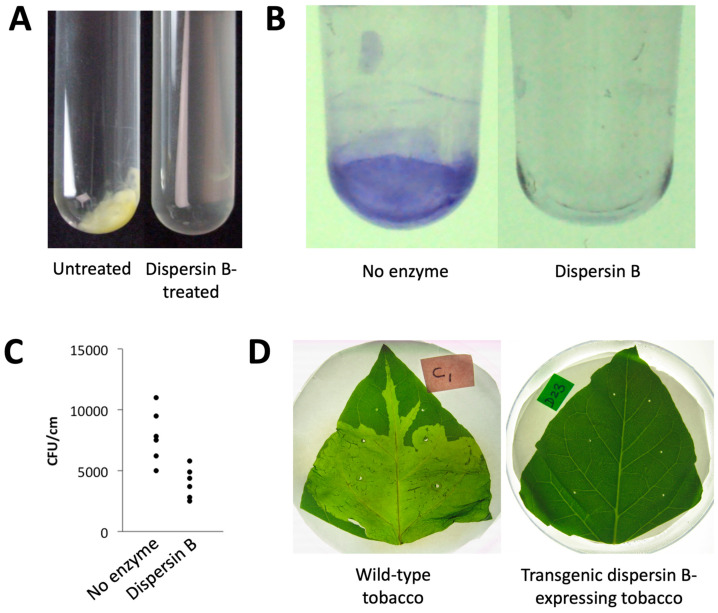
Effects of dispersin B on plant-associated bacteria: (**A**) *Xanthomonas citri* subsp. *citri* strain 306 forms aggregates when cultured in broth (**left panel**). These aggregates were rapidly dissolved upon dispersin B treatment (**right panel**). (**B**) Biofilm formation by *Ralstonia solanacearum* strain Molk2 in polystyrene microtiter plates in the absence or presence of 20 µg/mL dispersin B. Biofilms were stained with crystal violet. (**C**) Binding of *Pseudomonas fluorescens* strain WCS365 to tomato roots in the absence or presence of 20 µg/mL dispersin B. Bacteria were mixed with 6-day-old tomato roots for 90 min. The roots were then crushed, mixed by vortex agitation, diluted, and plated on agar for CFU enumeration. Each data point represents one individual root. (**D**) Tobacco leaves infected with *Pectobacterium carotovorum* subsp. *carotovorum* strain ATCC 15713. Leaves were photographed 24 h after inoculation: (**left**) wild-type tobacco leaf; (**right**) leaf from a transgenic tobacco plant expressing dispersin B. Source: (**A**–**C**) J.B. Kaplan, unpublished data; (**D**) Ragunath et al. [104], N. Ramasubbu, unpublished data.

**Antibiofilm activities of dispersin B against plant pathogens:** Dispersin B exhibits antibiofilm activity against several PNAG-producing plant pathogens, including members of the genera *Ralstonia*, *Xanthomonas*, and *Pectobacterium*, as well as the plant biocontrol bacterium *Pseudomonas fluorescens* (Figure 7). *X. citri* subsp. *citri*, the causative agent of citrus canker, forms aggregates when cultured in broth (Figure 7A). These aggregates are readily dissolved by dispersin B, suggesting that PNAG mediates intercellular adhesion in this species. Dispersin B also inhibited biofilm formation by *R. solanacearum* in polystyrene microtiter plates (Figure 7B). *R. solanacearum* is a causative agent of bacterial wilt in wide range of host plants. Dispersin B also inhibited biofilm formation by *Pseudomonas fluorescens* [101], as well as the binding of *P. fluorescens* planktonic cells to tomato roots (Figure 7C). Dispersin B also blocked biofilm formation by *P. carotovorum* in 96-well plates in vitro [104], as well as *P. carotovorum* infection of tobacco leaves in planta when *dspB* was expressed as a transgene (Figure 7D). Taken together, these findings suggest that plant-associated bacteria produce PNAG, and that PNAG contributes to intercellular adhesion, biofilm formation, plant colonization, and phytopathogenicity in vivo.

**In vivo studies:** Four different studies have demonstrated that dispersin B exhibits antibiofilm activity against staphylococci in vivo. Kaplan et al. [125] showed that dispersin B decreased the ability of *S. epidermidis* to colonize pig skin by 66–78% compared to a no-enzyme control. Gawande et al. [103] found that dispersin B combined with a silver wound dressing showed an 80% reduction in *S. aureus* MRSA bioburden in a chronic wound mouse model, compared to a 14% reduction when wounds were treated with a silver wound dressing alone. Darouiche et al. [61] tested dispersin B against *S. aureus* in a rabbit catheter infection model. Only 1 out of 30 catheters coated with dispersin B plus triclosan was colonized with *S. aureus*, compared to 29/30 uncoated control catheters. Finally, Serrera et al. [126] showed that dispersin B, when used in combination with teicoplanin as a catheter lock solution in a sheep model of port-related bloodstream infection, reduced the number of *S. aureus* infections from 100% to 50% (8/8 versus 4/8) and the number of deaths from 50% to 0% (4/8 versus 0/8), compared to a teicoplanin catheter lock solution alone.

## 7. Concluding Remarks

Dispersin B was licensed to the Canadian company Kane Biotech Inc. (Winnipeg, MB, Canada) in 2004, initially for the development of medical device coatings and cosmetics. Pharmaceutical-grade dispersin B (also known as DispersinB^®^) has been purified from a recombinant strain of *E. coli*, and the enzyme has undergone extensive biocompatibility testing. Recombinant dispersin B exhibited no cytotoxicity against L-929 cells in vitro and no mutagenicity or genotoxicity in the Ames test, in an in vitro human peripheral blood lymphocytes micronuclei assay, and in an in vivo rat blood reticulocyte assay that monitors chromosomal damage. Additional in vitro and in vivo biocompatibility testing showed that dispersin B was non-pyrogenic, non-sensitizing, and non-irritating, exhibited no acute or sub-chronic systemic toxicity, and was not detectable in blood when applied to full-thickness dermal wounds in pigs. These results suggest that dispersin B is biocompatible and safe for use on human skin. Dispersin B is also compatible with many antimicrobials, salts, preservatives, and excipients such as polyols, enabling it to be formulated into a plurality of products. For commercialization, dispersin B has been formulated into a gel containing poloxamer 407, glycerol, preservatives, and buffered phosphate. This formulation exhibits thermosensitive viscosity properties that enable it to form a gel on the skin. This dispersin B gel formulation has undergone stability and biocompatibility testing in accordance with ISO 10993 standards for prolonged exposure to breached/compromised skin, with positive results. Kane Biotech has also obtained promising results with dispersin B gel in a pig wound-healing study. The product has been designated as a biologic/device combination product, with the primary mode of action being the device. Dispersin B gel is currently undergoing the Investigational Device Exemption (IDE) review process with the FDA Center for Devices and Radiological Health (CDRH), with a plan to commence clinical trials for chronic wounds and acne vulgaris in 2025.

Dispersin B is a well-characterized PNAG-degrading enzyme that is both a useful tool for biofilm research and a potential therapeutic agent for the treatment and prevention of biofilm-related infections in plants and animals. Anti-PNAG antibodies have been shown to protect mice against local and/or systemic infections by various microbial pathogens, including *Streptococcus pyogenes*, *S. pneumoniae*, *Listeria monocytogenes*, *Neisseria meningitidis* serogroup B, *C. albicans*, and *Plasmodium berghei* ANKA, as well as against colonic pathology in a model of infectious colitis [38]. In addition, PNAG-based vaccines have been shown to be protective against a variety of PNAG-producing pathogens in animal models [39]. These findings validate PNAG as an antimicrobial target. Because of the large numbers of bacteria, fungi, and protozoa that produce PNAG [38,39], dispersin B may have applicability as a broad-spectrum antibiofilm agent. The most practical applications will be those where dispersin B can be used as a topical agent in the form of a gel, ointment, or spray in combination with an antimicrobial. These may include agents for the treatment of wounds, such as surgical site wounds, traumatic wounds, burns, and chronic wounds, including diabetic foot ulcers; for the treatment and prevention of dermatoses, such as atopic dermatitis and acne vulgaris; for the treatment of ocular infections, such as blepharitis and corneal ulcers; for the treatment of aural infections, such as otitis media; and as a pre-surgical skin antiseptic. Other potential applications include catheter lock solutions and irrigation solutions or coatings for implanted medical devices.

## Figures and Tables

**Table 1:** Orthologues of *A. actinomycetemcomitans dspB* in bacteria. Sequences were identified with a protein BLAST search using *A. actinomycetemcomitans* dispersin B (GenBank accession number WP_005566076) as a query sequence.

Species	Family	GenBank Accession No.	Amino Acids
*Actinobacillus capsulatus*	*Pasteurellaceae*	WP_018652103.1	378
*Actinobacillus equuli*	*Pasteurellaceae*	WP_039197353.1	378
*Actinobacillus lignieresii*	*Pasteurellaceae*	WP_126375001.1	377
*Actinobacillus pleuropneumoniae*	*Pasteurellaceae*	WP_005617581.1	377
*Actinobacillus succinogenes*	*Pasteurellaceae*	WP_012072607	508
*Actinobacillus suis*	*Pasteurellaceae*	WP_014991875.1	378
*Actinobacillus ureae*	*Pasteurellaceae*	WP_115607612.1	378
*Actinobacillus vicugnae*	*Pasteurellaceae*	WP_150540037.1	378
*Aggregatibacter actinomycetemcomitans*	*Pasteurellaceae*	WP_005566076	361
*Aggregatibacter aphrophilus*	*Pasteurellaceae*	OBY54997.1	403
*Aggregatibacter kilianii*	*Pasteurellaceae*	WP_275425143.1	339
*Basfia succiniciproducens*	*Pasteurellaceae*	WP_305367133	480
*Cardiobacterium hominis*	*Cardiobacteriaceae*	WP_281839854.1	528
*Exercitatus varius*	*Pasteurellaceae*	WP_317543108.1	508
*Haemophilus pittmaniae*	*Pasteurellaceae*	WP_269457014	381
*Kingella oralis*	*Neisseriaceae*	WP_315367803.1	405
*Lonepinella koalarum*	*Pasteurellaceae*	WP_228777406.1	363
*Mannheimia succiniciproducens*	*Pasteurellaceae*	AAU37718.1	501
*Neisseria animaloris*	*Neisseriaceae*	WP_199901419.1	517
*Neisseria brasiliensis*	*Neisseriaceae*	MRN37458.1	340
*Neisseria canis*	*Neisseriaceae*	WP_085415444.1	508
*Neisseria chenwenguii*	*Neisseriaceae*	WP_199720929.1	421
*Neisseria dentiae*	*Neisseriaceae*	WP_211276428.1	400
*Neisseria dumasiana*	*Neisseriaceae*	WP_085417823.1	395
*Neisseria montereyensis*	*Neisseriaceae*	WP_289623084.1	398
*Neisseria musculi*	*Neisseriaceae*	WP_187000616.1	388
*Neisseria oralis*	*Neisseriaceae*	WP_308022698.1	410
*Neisseria shayeganii*	*Neisseriaceae*	WP_220457298.1	770
*Neisseria wadsworthii*	*Neisseriaceae*	WP_009115775.1	468
*Neisseria weixii*	*Neisseriaceae*	WP_096294699.1	392
*Neisseria zalophi*	*Neisseriaceae*	WP_318527728.1	398
*Neisseria zoodegmatis*	*Neisseriaceae*	WP_085364538.1	395

**Table 2:** Antibiofilm activities of dispersin B against bacteria in vitro.

Species	Antibiofilm Activity	References
*Achromobacter xylosoxidans*	Inhibits biofilm formation; detaches preformed biofilms.	[82]
*Acinetobacter baumannii*	Inhibits “pellicle” formation at the air–liquid interface; inhibits biofilm formation; detaches preformed biofilms; sensitizes preformed biofilms to killing by antimicrobial peptide KSL-W.	[83,84]
*Actinobacillus pleuropneumoniae*	Inhibits biofilm formation; detaches preformed biofilms; sensitizes preformed biofilms to killing by ampicillin.	[14,15,50,85,86,87,88,89,90]
*Aggregatibacter actinomycetemcomitans*	Sensitizes planktonic cells to killing by human macrophages; sensitizes preformed biofilms to detachment by EDTA, SDS, proteinase K, and DNase; sensitizes preformed biofilms to killing by cetylpyridinium chloride and SDS; sensitizes preformed biofilms to killing by predatory *Bdellovibrio bacteriovorus* bacteria.	[91,92,93,94]
*Bordetella pertussis*, *B. parapertussis*	Inhibits biofilm formation; detaches preformed biofilms; sensitizes preformed biofilms to killing by antimicrobial peptides polymyxin B and LL-37.	[82,95,96,97]
*Burkholderia cepacia* complex	Inhibits biofilm formation; detaches preformed biofilms; sensitizes biofilms to killing by tobramycin.	[43,98]
*Corynebacterium striatum*	Detaches preformed biofilms.	[77]
*Cutibacterium acnes*	Inhibits surface attachment and biofilm formation; sensitizes biofilms to killing by benzoyl peroxide and tetracycline.	[99]
*Escherichia coli*	Inhibits biofilm formation; detaches preformed biofilms; sensitizes biofilms to killing by triclosan and bacteriophages.	[61,73,75,78,100,101]
*Francisella novicida*, *F. philomiragia*	Detaches preformed biofilms.	[102]
*Klebsiella pneumoniae*	Inhibits biofilm formation; sensitizes preformed biofilms to killing by antimicrobial peptide KSL-W.	[83,103]
*Pectobacterium atrosepticum, P. carotovorum*	Inhibits biofilm formation; detaches preformed biofilms.	[104,105]
*Pseudomonas fluorescens*	Inhibits biofilm formation; detaches preformed biofilms; inhibits attachment of planktonic cells to tomato roots.	[101]; J. B. Kaplan, unpublished data
*Ralstonia solanacearum*	Inhibits biofilm formation.	J. B. Kaplan, unpublished data
*Solobacterium moorei*	Inhibits biofilm formation.	[106]
*Staphylococcus aureus*	Inhibits biofilm formation; detaches preformed biofilms; sensitizes preformed biofilms to killing by triclosan, tobramycin, vancomycin, rifampicin, clindamycin, cefamandole nafate, and antimicrobial peptide KSL-W.	[60,61,83,103,107,108,109,110,111]
*Staphylococcus capitis*	Detaches preformed biofilms.	[112]
*Staphylococcus epidermidis*	Inhibits biofilm formation; detaches preformed biofilms; sensitizes preformed biofilms to killing by triclosan, cetylpyridinium chloride, ciprofloxacin, rifampicin, and antimicrobial peptide KSL-W.	[50,55,59,61,82,83,100,103,108,109,112,113,114,115,116,117,118]
*Staphylococcus pseudintermedius*	Inhibits biofilm formation; detaches preformed biofilms.	[119]
*Streptococcus mutans*	Detaches preformed biofilms; sensitizes preformed biofilms to detachment by oral irrigation.	[120]
*Streptomyces coelicolor*, *S. lividans*	Inhibits surface attachment and hyphal aggregation.	[121]
*Xanthomonas citri*	Disaggregates bacterial flocs (floating or suspended biofilms).	J. B. Kaplan, unpublished data
*Yersinia pestis*	Inhibits biofilm formation.	[101]

## Data Availability

No new data were created or analyzed in this study. Data sharing is not applicable to this article.
